# Stathmin 1 Expression in Neuroendocrine and Proliferating Prostate Cancer

**DOI:** 10.21203/rs.3.rs-5279702/v1

**Published:** 2024-12-09

**Authors:** Yingli Shi, Yunshin A. Yeh, Siyuan Cheng, Xin Gu, Shu Yang, Lin Li, Nazih P. Khater, Susan Kasper, Xiuping Yu

**Affiliations:** Louisiana State University Health Sciences Center at Shreveport; Overton Brooks VA Medical Center; Louisiana State University Health Sciences Center at Shreveport; Louisiana State University Health Sciences Center at Shreveport; Ochsner LSU Health; Louisiana State University Health Sciences Center at Shreveport; Louisiana State University Health Sciences Center at Shreveport; University of Cincinnati College of Medicine; Louisiana State University Health Sciences Center at Shreveport

## Abstract

Prostate cancer (PCa) is the second leading cause of cancer-related mortality among men in the United States. While PCa initially responds to androgen deprivation therapy, a significant portion progresses to castration-resistant PCa. Approximately 20–25% of these cases acquire aggressive neuroendocrine (NE) features, ultimately leading to neuroendocrine prostate cancer (NEPC). In this study, we used bioinformatics analysis, western blotting, and immunohistochemical staining to investigate the expression of stathmin 1 (STMN1) in PCa cell lines and tissue samples from human PCa and mouse models. Our findings revealed a correlation between elevated STMN1 expression, high Gleason Score, and poor clinical outcomes in PCa patients. Additionally, STMN1 expression was positively correlated with the cell proliferation marker Ki67. Importantly, we observed a significant increase in STMN1 expression in NEPC compared to prostate adenocarcinoma, suggesting its potential role as a diagnostic and prognostic marker for advanced PCa. Furthermore, elevated STMN1 expression was detected in TRAMP tumors, a mouse model of PCa, further supporting its association with PCa progression. In summary, our study highlights the increased expression of STMN1 in NEPC and proliferating prostate adenocarcinoma cells, indicating its potential utility as a diagnostic and prognostic marker for advanced PCa.

## Introduction

Prostate cancer (PCa) is the most commonly diagnosed solid tumor and the second leading cause of cancer-related deaths among men in the United States [1]. Despite advancements in treatment modalities, including surgery, radiation therapy, and androgen deprivation therapy (ADT), the clinical management of advanced PCa remains challenging. Although ADT initially suppresses tumor growth by targeting androgen receptor (AR) signaling, a significant proportion of prostate adenocarcinomas (AdPC) develop resistance to ADT, progressing to castration-resistant prostate cancer (CRPC). Notably, approximately 20–25% of CRPC cases acquire a neuroendocrine (NE) phenotype, an aggressive subtype of PCa characterized by rapid progression and poor prognosis [2].

The TRAMP (Transgenic Adenocarcinoma of the Mouse Prostate) model serves as a valuable tool for studying PCa progression. In TRAMP mice, a prostate-specific probasin promoter drives the expression of SV40 T-antigen specifically in the prostate, resulting in the development of prostatic intraepithelial neoplasia (PIN), with some lesions progressing to NEPC, a process accelerated by castration [3, 4].

Stathmin1 (STMN1) is an 18 kDa ubiquitous cytoplasmic protein that regulates microtubule dynamics. Accumulating evidence indicates that STMN1 expression is increased in various human malignancies, such as lung cancer [5], urinary bladder [6] and hepatocellular carcinoma [7]. Additionally, increased STMN1 expression has been reported in PIN and AdPC [8]. However, the expression levels of STMN1 in more aggressive forms of PCa, especially NEPC, and the underlying mechanisms for its elevation remain unexplored.

In this study, we evaluated STMN1 expression across various PCa tumor grades and different prostatic cell types. Our objective was to elucidate the role of STMN1 in aggressive PCa and assess its potential as a prognostic marker for advanced diseases. We found that elevated STMN1 expression correlated with high Gleason Scores, increased PCa cell proliferation, and poor clinical outcomes. Notably, STMN1 levels were significantly higher in NEPC compared to AdPC, suggesting that STMN1 may play a role in NEPC development.

## Materials and methods

### Bioinformatics analysis

STMN1 expression profiles and corresponding Gleason scores from the DFKZ datasets [9], as well as bulk RNA-seq data from patients with PCa (SU2C 2019 [10]) were retrieved from cBioPortal. In SU2C dataset, samples with an NEPC score > 0.4 and an AR score < 0.2 were considered NEPC samples. STMN1 expression profiles and Gleason scores from TCGA were acquired using the R package “TCGAbiolinks”. Bulk RNA-seq data from patients with neuroendocrine prostate cancer (Beltran Nat Med 2016 [11]) were downloaded from dbGaP using accession number phs000909. STMN1 expression levels were transformed via log2(TPM + 1) and compared between neuroendocrine prostate cancer (NEPC) and adenocarcinoma prostate cancer (AdPC), as well as across various Gleason scores. Heatmaps showing the expression of STMN1, PCNA, TOP2A, E2F1, and AR in the SU2C and Beltran datasets were generated using the “pheatmap” package in RStudio. Transcriptomic data for STMN1 expression across various PCa cell lines were extracted from the CTPC collection [12].

STMN1-associated genes (|Spearman’s correlation| > 0.5, p-value < 0.05) from the SU2C RNA-seq dataset was identified through cBioPortal. Gene ontology (GO) biological process annotation was performed using the “clusterProfiler” package in RStudio. Survival analysis and correlation analysis between STMN1 expression and Rb mutation status were conducted using cBioPortal online tools. Patients were stratified into two groups based on STMN1 expression levels: STMN1-high and STMN1-low. Kaplan-Meier survival analysis was performed to assess overall survival.

Data on STMN1 expression in normal neuroendocrine (NE) cells were extracted from the HuPSA single-cell RNA sequencing dataset collection [13], which includes samples from wild-type mice, with NE cells identified using the HuPSA pipeline.

### Human and murine PCa sample collection

A total of 71 human prostatic specimens were utilized in this study, obtained from Overton Brook VA medical Center, Louisiana State University Health Sciences Center at Shreveport Biorepository Core, and Tissue for Research, as described previously [14]. The specimens were classified by pathologists based on histology, Gleason grades, and the expression of NEPC biomarker, insulinoma-associated-1, INSM1. These samples include benign prostate (n = 13), Gleason score 3 + 3 (n = 5), GS 3 + 4 (n = 11), GS 4 + 3 (n = 11), GS 4 + 4 (n = 8), GS 4 + 5 (n = 8), GS 5 + 4 (n = 4), GS 5 + 5 (n = 3), and NEPC tissues (n = 8). Archived TRAMP tumor sections, including both intact and castrated samples, were also used in this study. All samples were collected and utilized in accordance with protocols approved by the Institutional Animal Care and Use Committee and the Institutional Review Board of LSU Health Shreveport.

### Cell culture and Western blotting analysis

PCa cell lines (VCaP, LNCaP, C42B, 22RV1, PC3, and DU145) were cultured in RPMI 1640 medium supplemented with 10% fetal bovine serum and antibiotics (penicillin and streptomycin) at 37°C in a humidified 5% CO2 incubator. NCI-H660 cells were grown *in vivo* as xenograft tumors. Total proteins were extracted from PCa cells and xenograft tissues using CelLytic M cell lysis reagent (Sigma-Aldrich). Western blot analysis was performed following established protocols [14]. Antibodies against STMN1 (13655S) were obtained from Cell Signaling Technology (Beverly, MA), and antibodies against E2F1 (sc-251) and beta-actin (sc-47778) were purchased from Santa Cruz Biotechnology (Dallas, TX).

### Immunohistochemistry (IHC) and Immunofluorescence (IF) staining

Tissue sections (5-^m thick) were prepared from formalin fixed paraffin-embedded specimens, and IHC was performed following established protocols [14]. INSM1 (insulinoma-associated protein 1, sc-271408) from Santa Cruz Biotechnology (Dallas, TX) was used as primary antibody. STMN1 expression was assessed using the Allred scoring system, which combines a Proportion Score (PS) and an Intensity Score (IS) to generate a total score ranging from 0 to 8. The PS was determined based on the percentage of positively stained cells: 0 (0%), 1 (< 1%), 2 (1–10%), 3 (11–33%), 4 (34–66%), and 5 (67–100%). The IS was scored as 0 (negative), 1+ (weak), 2+ (moderate), and 3+ (strong). The total score was calculated by summing the PS and IS.

IF staining was performed according to previous protocols [14]. The primary antibodies used for IF staining included Ki67 (9449S, Cell Signaling Technology, Beverly, MA), androgen receptor (AR, sc-816, Santa Cruz Biotechnology, Dallas, TX), SV40 T antigen (TAg, sc-147, Santa Cruz Biotechnology, Dallas, TX), chromogranin A (CHGA, CPTC-CHGA-1, DSHB, Iowa City, IA), and synaptophysin (SYP, 611880, BD Biosciences).

### Statistical analysis

Group comparisons were performed using the Wilcoxon rank-sum test and the Kruskal-Wallis test, followed by post-hoc Dunn’s test. The prognostic impact of low versus high STMN1 expression was evaluated using the Kaplan-Meier method, with survival differences analyzed using the log-rank test. Correlations between STMN1 expression and Ki67 (or E2F1), as well as between the STMN1 Allred score and Gleason score, were assessed using the Spearman correlation test. A p-value of < 0.05 was considered statistically significant.

## Results

### STMN1 expression is higher in NEPC compared to AdPC

**Figure 1:**
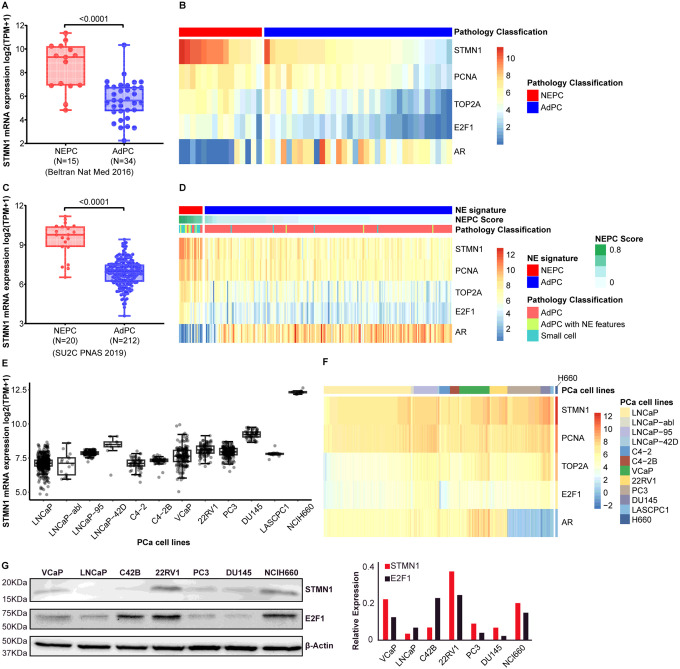
Expression profile of stmn1 in prostate cancer (PCa). The mRNA expression data of STMN1 in human PCa patients were extracted from Beltran Nat Med 2016 dataset (A & B) and SU2C PNAS 2019 study (C & D). The mRNA expression of STMN1 was significantly higher in neuroendocrine prostate cancer (NEPC) compared to adenocarcinoma prostate cancer (AdPC) (P<0.01). Heatmaps were generated to illustrate the expression of STMN1, androgen receptor (AR) and cell proliferation markers including PCNA, TOP2A and E2F1 in NEPC versus AdPC. The mRNA expression data of STMN1 across PCa cell lines were extracted from the CTPC collection. (G) Western blot analysis confirmed the protein expression of STMN1 and E2F1 in PCa cell lines, with the right panel displaying relative quantification of western blot results.

STMN1 expression was analyzed using cohorts that contain both AdPC and NEPC samples, including the Neuroendocrine Prostate Cancer dataset (Beltran Nat Med 2016 [11] and SU2C 2019 [10]). The analysis revealed a significant increase in STMN1 expression in NEPC compared to AdPC tumors (Figs. 1A and 1C, p < 0.001). Heatmaps were generated to visualize the expression of STMN1 along with marker genes of cell proliferation (Figs. 1B and 1D). These heatmaps illustrated elevated STMN1 levels in NEPC, along with increased expression of cell proliferation markers such as PCNA and TOP2A. In contrast, AR mRNA expression was lower in NEPC compared to AdPC specimens, consistent with NEPC characteristics, as lower AR expression is a hallmark of NEPC.

Additionally, STMN1 expression in PCa cell lines representing both AdPC and NEPC was examined using RNA-seq data collected in the CTPC study [12]. The NEPC cell line NCI-H660 exhibited the highest STMN1 mRNA expression compared to AR-positive AdPC lines (LNCaP, LNCaP-abl, LNCaP-95, LNCaP-42D, C4–2, C4–2B, VCaP and 22RV1) as well as AR-negative AdPC lines (DU145, PC3 and LASPCP1) (Fig. 1E). In NCI-H660 cells, elevated STMN1 mRNA expression was accompanied by increased mRNA levels of PCNA, TOP2A, and E2F1, along with decreased AR mRNA expression compared to AdPC cell lines (Fig. 1F).

To validate these *in-silico* findings, Western blotting analysis of PCa cell lines was conducted on PCa cell lines. Notable STMN1 protein expression was detected in NCI-H660 cells. Interestingly, significant STMN1 protein levels were also observed in 22RV1 and VCaP cell lines, which differed from the mRNA expression patterns in these cells. Lower levels of STMN1 protein were found in DU145, PC3, and C4–2B cells, with STMN1 being barely detectable in LNCaP cells (Fig. 1G).

### Elevated STMN1 mRNA expression correlates with higher Gleason score and predicts poor overall survival

**Figure 2:**
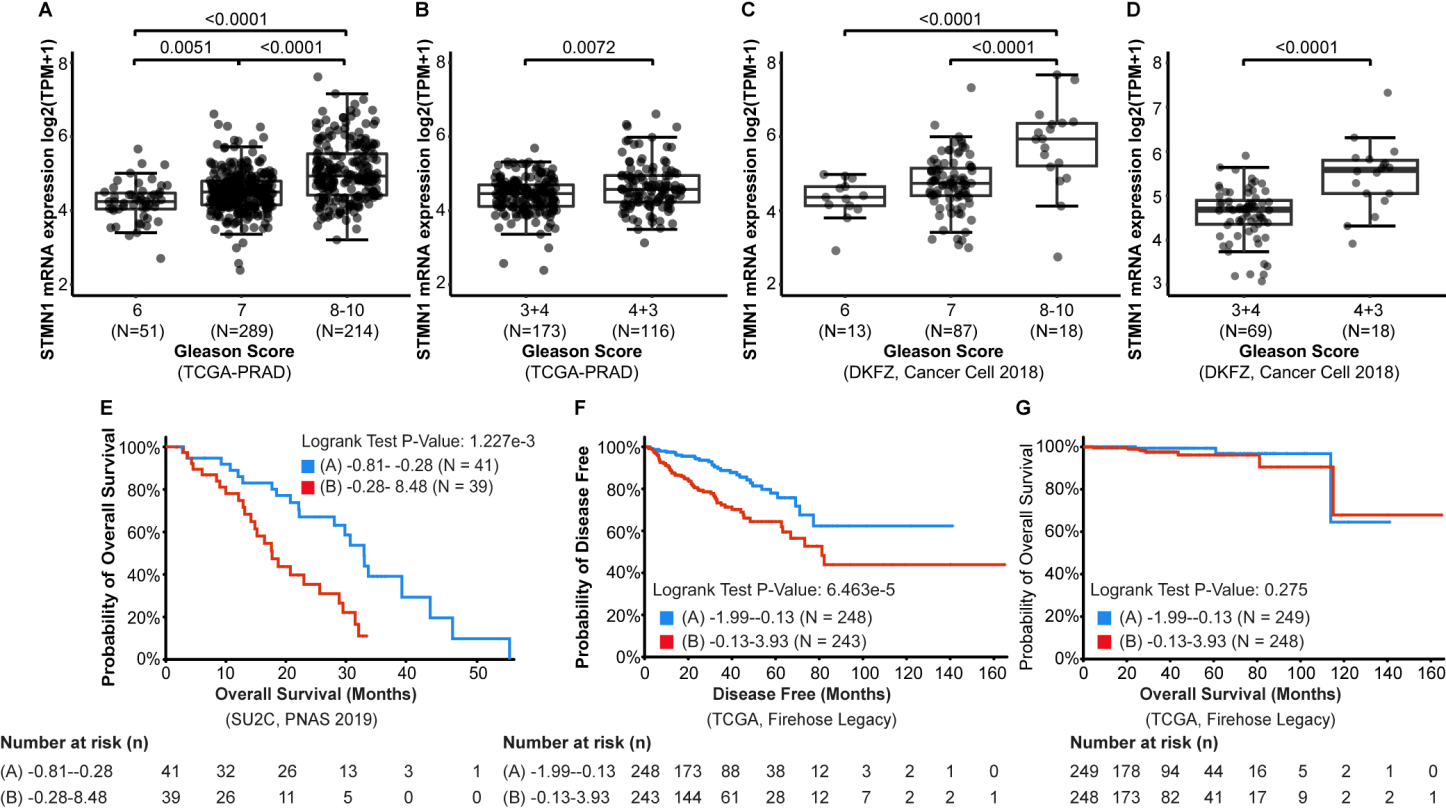
Association of STMN1 expression with tumor grade and clinical outcomes in prostate cancer (PCa). Gene expression and clinical data were extracted from the TCGA-PRAD (A & B) and DFKZ (C & D) datasets. (A & C) STMN1 expression levels were significantly higher in high-grade tumors (GS ≥ 8) compared to low-grade (GS 6) and intermediate-grade tumors (GS 7) in both TCGA-PRAD and DFKZ datasets. (B & D) STMN1 expression was significantly higher in the GS 4+3 group compared to the GS 3+4 group in both datasets. (E & F) Kaplan-Meier survival analysis of the SU2C (PNAS, 2019) and TCGA (Firehose Legacy) datasets showed significantly shorter overall survival and disease-free survival, respectively, in patients with high STMN1 expression compared to those with low STMN1 expression (p < 0.01). (G) No significant difference in overall survival was observed between high and low STMN1 expression groups in the TCGA (Firehose Legacy) dataset.

STMN1 expression was analyzed across various grades of PCa, ranging from low to high grade, using data from the TCGA-PRAD and DKFZ datasets. As expected, STMN1 expression was highest in high-grade tumors ((Gleason Score (GS) 8–10)) compared to low and intermediate- grade tumors (GS 6 and 7; p < 0.01) in both datasets (Figs. 2A and 2C). Given the clinical significance of Gleason Score in prognosis, specifically, that GS 4 + 3 is considered more aggressive and carries a higher risk of progression to metastatic PCa compared to GS 4 + 3 [15], STMN1 expression was compared between these two groups in both datasets as well. Results showed that STMN1 expression was significantly higher in the GS 4 + 3 group compared to the GS 3 + 4 group (p < 0.01, Figs. 2B and 2D).

Furthermore, the association between STMN1 expression and clinical outcomes was also explored. In the SU2C dataset, patients with high STMN1 expression exhibited a lower overall survival rate compared to those with low STMN1 expression (p < 0.01; Fig. 2E). In TCGA dataset, although there was no significant difference in overall survival rates between the STMN1-high and -low patients (p = 0.41; Fig. 2G), patients with high STMN1 expression had a significantly lower probability of disease-free survival compared to the low expression group (p < 0.01; Fig. 2F).

### Increased STMN1 protein expression is associated with advanced AdPC and NEPC

**Figure 3:**
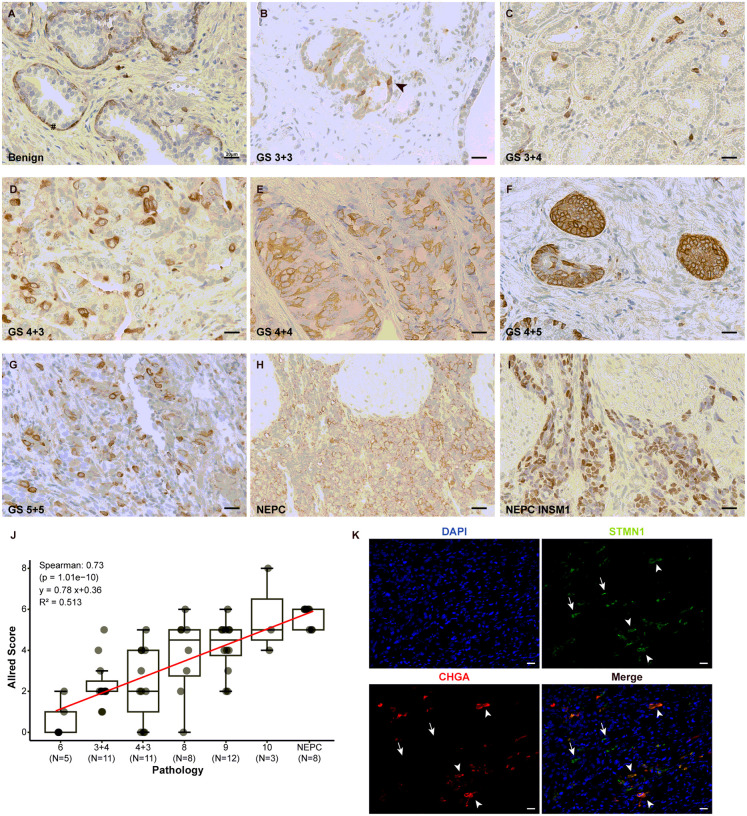
The expression of STMN1 protein in human prostatic tissues. (A-H) Representative immunohistochemical staining of STMN1 in human prostatic tissues. (A) Positive STMN1 staining was observed in basal epithelial cells in benign prostatic tissue. (B-G) Varying intensity of STMN1 staining were present in luminal and/or basal epithelial cells in adenocarcinoma tissues. The # symbol and arrowhead indicated basal epithelium cells and luminal epithelium cells, respectively. (H & I) Serial sections of a NEPC tumor showed STMN1 expression in the NEPC area (highlighted by the expression of NEPC marker, INSM1) but no expression was detected in adjacent AdPC cells. (J) Distribution of STMN1 expression (Allred score) among prostate specimens, demonstrating an association between STMN1 expression and Gleason Score of PCa. (Spearman correlation: 0.73, p < 0.01). (K) Dual immunofluorescence staining to access the co-expression STMN1 with NEPC marker Chromogranin A (CHGA) in AdPC tumor with NE differentiation. STMN1 expression was detected in both NE (arrowheads) and non-NE (arrows) cells. Scale bars =20 μm.

STMN1 protein expression was accessed through IHC staining in tissue sections from benign prostate, AdPC and NEPC. In benign prostate tissues, STMN1 staining was present in basal epithelial cells but absent in luminal epithelial cells across all the benign tissues examined (Fig. 3A, n = 13), consistent with previous reports [8].

In AdPC and NEPC, STMN1 protein expression was quantified using Allred scores. In all GS 6 and 81% of the GS 3 + 4 cases, STMN1 expression was undetectable or minimal (Allred Score < 3). Weak STMN1 expression (Allred score 3–4) was observed in 36.3% of GS 4 + 3 cases, while moderate to high expression (Allred Score > 4) was noted in 56% of GS 8–10 samples. A strong positive correlation was found between STMN1 expression and PCa pathology (Spearman correlation: 0.73, p < 0.01, Fig. 3J).

In contrast, all NEPC cases (n = 8) exhibited moderate to strong STMN1 expressions (Allred score > 4, Figs. 3H and 3J). IHC staining with an anti-INSM1 antibody highlighted the NEPC areas in serial sections (Fig. 3I). Furthermore, NEPC cells scattered in an AdPC tumor with neuroendocrine differentiation also displayed high STMN1 expression, as indicated by dual immunofluorescence staining for the NE marker Chromogranin A (CHGA) and STMN1 (Fig. 3K).

Collectively, RNA-seq, WB and IHC analyses demonstrate that increased STMN1 expression is strongly associated with higher Gleason scores. Notably, STMN1 expression is significantly elevated in NEPC compared to AdPC, and this heightened expression correlates with poorer overall survival.

### STMN1 is expressed in neuroendocrine cells in normal and benign prostate

**Figure 4:**
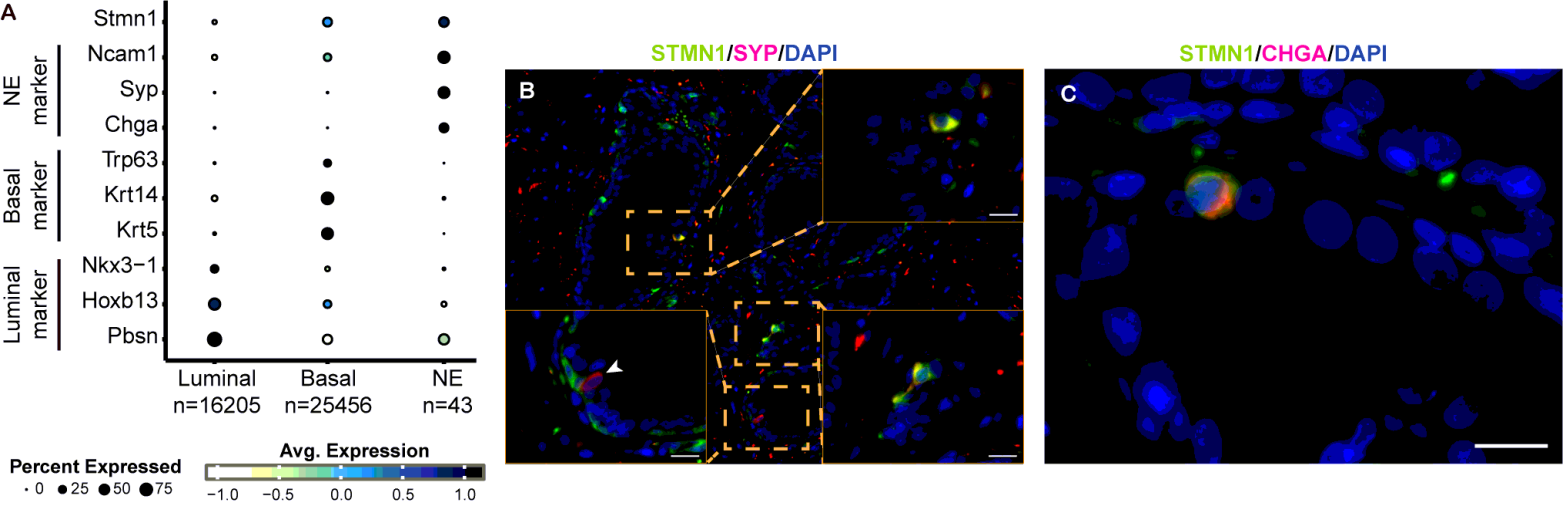
Stmn1 expression in normal neuroendocrine (NE) cells. (A) Stmn1 expression in normal NE cells in murine prostate. Single-cell RNA sequencing data from prostates of wild-type mice were obtained from Sawyers’ study collected in MoPSA [13, 16]. Specific markers used to identify different cell types include Nkx3-1, Hoxb13, and Pbsn for luminal epithelial cells; Krt5, Krt14, and Trp63 for basal epithelial cells; and Chga, Syp, and Ncam1 for NE cells. (B & C) Dual immunofluorescence staining showing co-expression of STMN1 with NE markers CHGA or SYP in human benign prostate specimens. STMN1 expression was detected in cells positive for SYP or CHGA expression. Arrowhead in panel B indicated a STMN1-negative NE cell. Scale bars =20 μm.

Capturing normal NE cells in prostatic tissue samples proved challenging due to the limited availability of prostate specimens from healthy individuals and the extremely low number of NE cells in normal prostatic tissues. To gain insight into STMN1 expression in normal NE cells, single-cell RNA sequencing (scRNA-seq) data from wild-type mice were utilized [13, 16]. Analysis of a total of 85,291 cells from wild type murine prostate revealed that normal NE cells constituted approximately 0.05% of the total cell population. Of note was that Stmn1 expression was enriched in normal NE cells, along with other NE markers Ncam1, Syp, and Chga, compared to luminal or basal epithelial cells (Fig. 4A).

To assess STMN1 expression in NE cells within human benign prostatic tissues, dual immunofluorescence staining was performed with STMN1 and the NE markers Chromogranin A (CHGA) or Synaptophysin (SYP). NE cells co-expressing STMN1 and the NE markers SYP and CHGA were detected (Figs. 4B & 4C). Notably, one NE cell exhibited negative STMN1 staining (arrowhead in Fig. 4B).

Collectively, STMN1 expression was detected in NE cells of both murine normal prostate tissues and human benign prostate tissues.

### STMN1 expression correlates with a proliferative phenotype

**Figure 5:**
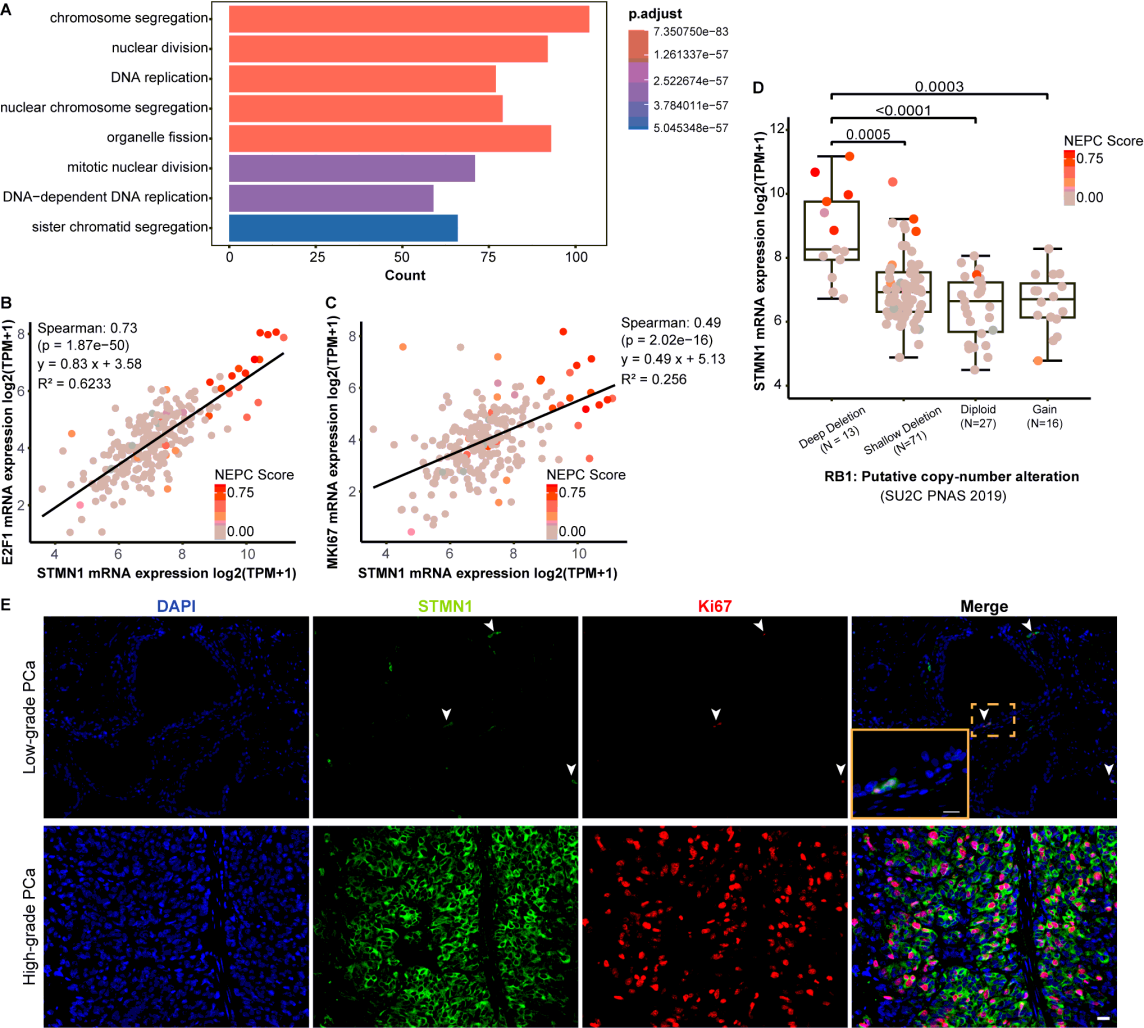
Correlation of STMN1 expression with cell proliferation in PCa. (A) Gene ontology (GO) analysis revealed pathways enriched in STMN1-positive PCa. (B & C) STMN1 expression positively correlates with Ki67 (B) and E2F1 (C) in human PCa (SU2C dataset, PNAS 2019) (p<0.01). (D) STMN1 expression was significantly higher in RB1-deleted PCa group compared to the diploid group, based on data extracted from SU2C 2019 dataset. (E) Dual immunofluorescence staining to show the co-expression of STMN1 in Ki67-positive cells in AdPC. The arrowhead denoted cells co-expressed Ki67 and STMN1. Scale bar = 20 μm.

Genes associated with STMN1 expression in PCa were identified in the SU2C 2019 cohort, resulting in a total of 362 STMN1-correlated genes (|spearman’s correlation| > 0.5 and p-value< 0.05). Gene Ontology (GO) analysis revealed that these STMN1-correlated genes were significantly enriched in biological processes such as “chromosome segregation”, “nuclear division”, “DNA replication” and “mitotic nuclear division” (Fig. 5A), supporting a role of STMN1 in cell proliferation.

Consistent with this, significant correlations were observed between STMN1 expression and the cell proliferation marker Ki67 (Fig. 5B, p < 0.01) as well as cell cycle regulator E2F1 (Fig. 5C, p < 0.01) in PCa.

Analysis of RB1, a key regulator of cell proliferation that is often deficient in PCa, indicated that STMN1 mRNA expression was higher in specimens with RB1 deep deletions compared to those with shallow deletions, RB1-diploid or gain groups (Fig. 5D, p < 0.01).

Additionally, dual IF staining revealed the co-expression of STMN1 with Ki67 in luminal cells in both benign and adenocarcinoma prostatic tissues (Figs. 5E), reinforcing the association of STMN1 expression with a proliferative phenotype.

### Stmn1 expression is associated with NE and cell proliferation in TRAMP model

**Figure 6:**
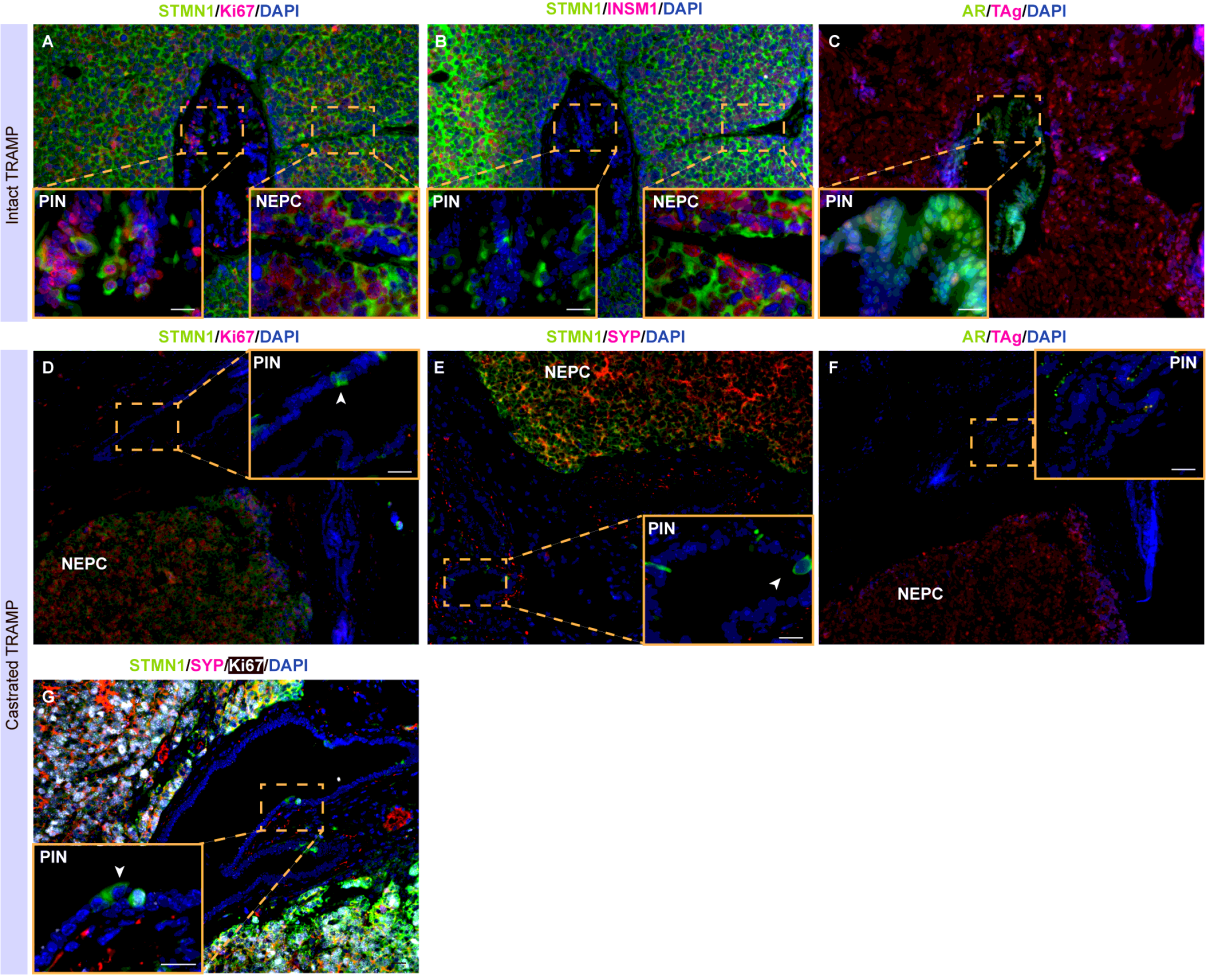
Immunofluorescence (IF) staining of STMN1 in TRAMP tumors from intact (A-C) and castrated mice (D-G). (A-C) In intact TRAMP tumors, high STMN1 expression was detected in both NEPC areas (approximately 100% of NEPC cells, indicated by the co-expression of INSM1) and adjacent PIN lesions (over 50% of PIN cells, which lacked INSM1 expression). While TAg was expressed in both PIN and NEPC cells, notable AR protein was detected in PIN cells but not in NEPC cells (C). In castrated TRAMP tumors, STMN1 expression was detected in almost all NEPC cells but was largely absent in PIN cells (D-F). Rare Stmn1-positive cells in the PIN lesions did not express Ki67 (D), SYP (E), or TAg (F), indicating the presence of non-proliferating, non-NE cells that express Stmn1. (G) Triple IF staining for STMN1/SYP/Ki67 revealed co-expression of STMN1 with Ki67 and SYP in NEPC. Some rare STMN1-positive cells in the PIN lesions did not express SYP nor Ki67. The arrowhead denotes a STMN1-positive cell that was neither proliferating nor NE. N>3 for each group. Scale bar = 20 μm

The transgenic adenocarcinoma of the mouse prostate (TRAMP) model, which mimics the development of human NEPC, was used to investigate the correlation between STMN1 expression and NEPC development [4]. Prostatic tumors containing intraepithelial neoplasia (PIN) and NEPC lesions from both intact and castrated mice were analyzed. In these samples, NEPC cells exhibited high levels of STMN1, T-antigen (TAg), Ki67, and NE markers including INSM1 and SYP, while lacking AR expression. In contrast, PIN lesions in intact mice expressed STMN1, AR, TAg and Ki67. However, in castrated TRAMP mice, expressions of STMN1, Ki67, AR and TAg were largely absent in PIN cells (Figs. 6A–6G), with only a few rare PIN cells (< 5%) showing STMN1 expression (Figs. 6D–6E). Notably, some STMN1 + PIN cells lacked Ki67, TAg or NE marker SYP expression (arrowhead in Fig. 6G).

Taken together, these observations demonstrate that STMN1 expression is associated with cell proliferation and the NE phenotype, independent of androgen status.

### STMN1 is the predominant isoform in PCa

**Figure 7:**
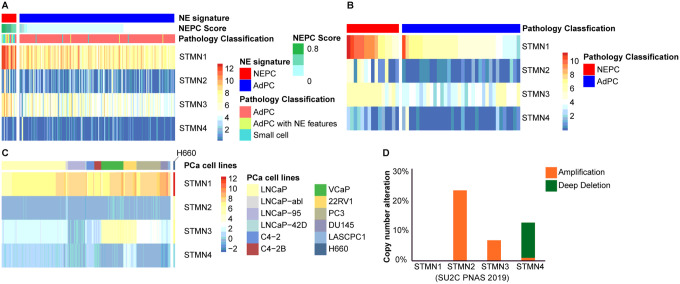
Differential expression of STMN family members in PCa. (A & B) Heatmap showing the expression levels of STMN family members in patient samples. STMN1 was the predominant isoform, followed by STMN3. Both STMN1 and STMN3 mRNA levels were significantly higher in NEPC compared to AdPC (p<0.01). Gene expression data were extracted from two RNA-seq datasets SU2C (A) and Beltran (B). (C) Heatmap of STMN family members in PCa cell lines. RNA-seq data were extracted from CTPC collection. (D) Copy number alterations of STMN family members in PCa, based on SU2C cohorts.

Finally, *in-silico* analyses were conducted using the SU2C [10], Beltran [11] cohorts, and CTPC collection [12] to identify the predominant STMN family member in PCa, including both patients samples and various PCa cell lines. As shown in Fig. 7, STMN1 was the most highly expressed STMN member in PCa patient specimens, followed by STMN3, while STMN2 and STMN4 levels were low. Notably, STMN3 expression was significantly higher in NEPC compared to AdPC (p < 0.01), paralleling the expression patterns observed for STMN1. High levels of STMN1 were also observed in NEPC NCIH660 cells (Figs. 7C).

Importantly, STMN1 shows a lower rate of copy number alterations (< 1%) compared to other STMN family members in SU2C cohort (Fig. 7D). STMN2 gene exhibited amplification in 24% of cases, and STMN3 showed amplification in 7%. Interestingly STMN4 had a deep deletion in 12% of SU2C samples (Fig. 7D).

## Discussion

NEPC is an aggressive subtype of PCa characterized by poor prognosis and limited therapeutic options [17]. This study investigated the expression of STMN1, a microtubule-destabilizing protein, in NEPC, revealing its significantly elevated expression in NEPC compared to AdPC. Our findings, drawn from extensive analyses of multiple datasets, IHC, and IF staining in both human and murine specimens, highlight STMN1’s potential as a diagnostic marker and therapeutic target for this challenging subtype of PCa. Importantly, our results indicate that STMN1 is not only expressed in NEPC but also in normal NE cells, suggesting its involvement in the NE phenotype of prostatic cells. Moreover, the elevated expression of STMN1 observed in proliferating PCa cells and its positive correlation with tumor grades and poor clinical outcomes underscore its potential as a prognostic marker in PCa. This finding aligns with previous studies indicating that the expression of STMN1 is upregulated in proliferating cancer cells [18].

A key finding of this study is the correlation between STMN1 expression and the loss of RB1, a critical tumor suppressor that regulates the cell cycle by binding to E2Fs and blocking their transcriptional activation [19]. In advanced PCa, RB1 genomic alterations have been linked to poor prognosis [10], particularly in CRPC and NEPC, where RB1 loss exceeds 70% [20, 21]. Our study revealed a positive correlation between elevated STMN1 expression and loss of RB1 (or increased E2F1 expression) in PCa, suggesting a potential crosstalk between the RB/E2F1 axis and STMN1 regulation in PCa, particularly in the context of NEPC development. Supporting this, prior research has shown that E2F1 can transactivate STMN1 expression in hepatocellular carcinoma [22], further supporting the regulatory relationship between the RB/E2F1 axis and STMN1.

Moreover, the expression patterns of STMN1 in the TRAMP mouse model further corroborate this relationship, highlighting the potential impact of RB1 inactivation on STMN1 expression. TRAMP is a T-antigen transgenic mouse model where T-antigen expression is driven by an androgen-responsive probasin promoter. In intact TRAMP mice, T-antigen is expressed in both PIN and NEPC cells. However, in castrated TRAMP mice, T-antigen expression is absent in PIN cells due to inactive androgen receptor (AR) signaling but persists in NEPC cells via the induction of Foxa2 [23]. Interestingly, STMN1 expression shows a similar pattern to that of T-antigen in TRAMP tumors, it is present in PIN cells of intact mice, rarely in PIN cells of castrated mice, and consistently in NEPC under both intact and castration conditions. Given that T-antigen inactivates RB1, the inactivation of RB1 could be a mechanism to activate E2F1, subsequently inducing the expression of STMN1. This further supports the involvement of the RB/E2F1 axis in regulating STMN1 expression in PCa, particularly in the context of NEPC development.

Interestingly, in the PIN lesions of castrated TRAMP mice, we observed rare cells that express STMN1 but are negative for T-antigen, Ki67 and Synaptophysin (Fig. 6G). What cell types are these Stmn1-positive, T-antigen negative, non-NE, and non-proliferative cells? Previous research has indicated that STMN1 is expressed in neural progenitor cells [24]. This raises intriguing possibilities that these cells could represent neuro-progenitor cells or early-stage trans-differential cells, potentially contributing to cancer progression or resistance mechanisms.

During cell proliferation, STMN1 plays a key role in maintaining “microtubule dynamics”, which are essential for mitotic phase entry and exit. Taxanes, such as paclitaxel and docetaxel, disrupt microtubule dynamics, leading to cell apoptosis [25]. Studies have shown that STMN1 mediates resistance to anti-mitotic chemo-drugs in tumor cells [5] and its inhibition enhances sensitivity to paclitaxel/docetaxel in osteosarcoma [26], gastric cancer [27] and PCa [28]. Given its association with resistance to anti-mitotic therapies, elevated STMN1 expression in advanced PCa raises important questions regarding the efficacy of Taxane-based chemotherapy. Our data suggest that high STMN1 expression may serve as a clinical indicator of poor response to these treatments and that targeted strategies to inhibit STMN1 could potentially improve therapeutic outcomes for patients with advanced PCa.

In conclusion, our study presents compelling evidence that STMN1 is a significant marker of aggressive PCa phenotypes, including NEPC. Its association with increased proliferation and poorer survival positions STMN1 as a promising candidate for further investigation as both a biomarker and a therapeutic target.

## Data Availability

Data Availability StatementAll public data listed in the “Methods” section can be assessed through cBioportal (https://www.cbioportal.org/), dbGaP(https://www.ncbi.nlm.nih.gov/gap/), CTPC (https://pcatools.shinyapps.io/CTPC_dev/) and HuPSA MoPSA (https://pcatools.shinyapps.io/HuPSA-MoPSA/). Data sharing is not applicable to this article as no datasets were generated during the current study.

## References

[1] Siegel RL, Cancer statistics. 2022. CA Cancer J Clin. 2022;72:7–33. 10.3322/caac.21708 35020204

[2] Davies AH. Cellular plasticity and the neuroendocrine phenotype in prostate cancer. Nat Rev Urol. 2018;15:271–86. 10.1038/nrurol.2018.22. 29460922

[3] Huss WJ. Origin of androgen-insensitive poorly differentiated tumors in the transgenic adenocarcinoma of mouse prostate model. Neoplasia. 2007;9:938–50. 10.1593/neo.07562. 18030362 PMC2077885

[4] Cacciatore A. Preclinical models of neuroendocrine prostate cancer. Curr Protoc. 2023;3:e742. 10.1002/cpz1.742. 37166213

[5] Bao P. High stmn1 expression is associated with cancer progression and chemo-resistance in lung squamous cell carcinoma. Ann Surg Oncol. 2017;24:4017–24. 10.1245/s10434-017-6083-0. 28933054

[6] Hemdan T. The prognostic value and therapeutic target role of stathmin-1 in urinary bladder cancer. Br J Cancer. 2014;111:1180–7. 10.1038/bjc.2014.427. 25072257 PMC4453855

[7] Liu J. Aberrantly high activation of a foxm1-stmn1 axis contributes to progression and tumorigenesis in foxm1-driven cancers. Signal Transduct Target Ther. 2021;6:42. 10.1038/s41392-020-00396-0. 33526768 PMC7851151

[8] Ghosh R. Increased expression and differential phosphorylation of stathmin may promote prostate cancer progression. Prostate. 2007;67:1038–52. 10.1002/pros.20601. 17455228

[9] Gerhauser C. Molecular evolution of early-onset prostate cancer identifies molecular risk markers and clinical trajectories. Cancer Cell. 2018;34:996–e10118. 10.1016/j.ccell.2018.10.016. 30537516 PMC7444093

[10] Abida W. Genomic correlates of clinical outcome in advanced prostate cancer. Proc Natl Acad Sci U S A. 2019;116:11428–36. 10.1073/pnas.1902651116. 31061129 PMC6561293

[11] Beltran H. Divergent clonal evolution of castration-resistant neuroendocrine prostate cancer. Nat Med. 2016;22:298–305. 10.1038/nm.4045. 26855148 PMC4777652

[12] Cheng S. Ctpc, a combined transcriptome data set of human prostate cancer cell lines. Prostate. 2023;83:158–61. 10.1002/pros.24448. 36207780 PMC9771918

[13] Cheng S. Unveiling novel double-negative prostate cancer subtypes through single-cell rna sequencing analysis. NPJ Precis Oncol. 2024;8:171. 10.1038/s41698-024-00667-x. 39095562 PMC11297170

[14] Cheng S. The expression of yap1 is increased in high-grade prostatic adenocarcinoma but is reduced in neuroendocrine prostate cancer. Prostate Cancer Prostatic Dis. 2020;23:661–9. 10.1038/s41391-020-0229-z. 32313141 PMC7572469

[15] Stark JR. Gleason score and lethal prostate cancer: Does 3 + 4 = 4 + 3? J Clin Oncol. 2009;27:3459–64. 10.1200/JCO.2008.20.4669. 19433685 PMC2717753

[16] Karthaus WR. Regenerative potential of prostate luminal cells revealed by single-cell analysis. Science. 2020;368:497–505. 10.1126/science.aay0267. 32355025 PMC7313621

[17] Yamada Y. Clinical and biological features of neuroendocrine prostate cancer. Curr Oncol Rep. 2021;23:15. 10.1007/s11912-020-01003-9. 33433737 PMC7990389

[18] Rana S. Stathmin 1: A novel therapeutic target for anticancer activity. Expert Rev Anticancer Ther. 2008;8:1461–70. 10.1586/14737140.8.9.1461. 18759697

[19] Chinnam M. Rb1, development, and cancer. Curr Top Dev Biol. 2011;94:129–69. 10.1016/B978-0-12-380916-2.00005-X. 21295686 PMC3691055

[20] Tan H-L. Rb loss is characteristic of prostatic small cell neuroendocrine carcinoma. Clin Cancer Res. 2014;20:890–903. 24323898 10.1158/1078-0432.CCR-13-1982 PMC3931005

[21] Beltran H. Divergent clonal evolution of castration-resistant neuroendocrine prostate cancer. Nat Med. 2016;22:298–305. 26855148 10.1038/nm.4045 PMC4777652

[22] Chen YL. The e2f transcription factor 1 transactives stathmin 1 in hepatocellular carcinoma. Ann Surg Oncol. 2013;20:4041–54. 10.1245/s10434-012-2519-8. 22911364

[23] Connelly ZM. Foxa2 activates the transcription of androgen receptor target genes in castrate resistant prostatic tumors. Am J Clin Exp Urol. 2018;6:172–81. 30510969 PMC6261871

[24] Wang W. Transcriptome dynamics of hippocampal neurogenesis in macaques across the lifespan and aged humans. Cell Res. 2022;32:729–43. 10.1038/s41422-022-00678-y. 35750757 PMC9343414

[25] Morse DL. Docetaxel induces cell death through mitotic catastrophe in human breast cancer cells. Mol Cancer Ther. 2005;4:1495–504. 10.1158/1535-7163.MCT-05-0130. 16227398

[26] Wang R. Inhibiting proliferation and enhancing chemosensitivity to taxanes in osteosarcoma cells by rna interference-mediated downregulation of stathmin expression. Mol Med. 2007;13:567–75. 10.2119/2007-00046.Wang. 17873971 PMC1976861

[27] Meng ZJ. Enhancement of chemosensitivity by stathmin-1 silencing in gastric cancer cells in situ and in vivo. Oncol Res. 2016;23:35–41. 10.3727/096504015X14452563486057. 26802649 PMC7842403

[28] Mistry SJ. Therapeutic interactions between stathmin inhibition and chemotherapeutic agents in prostate cancer. Mol Cancer Ther. 2006;5:3248–57. 10.1158/1535-7163.MCT-06-0227. 17172428

